# Fabrication and *in-vitro* Evaluation of Ketotifen Fumarate-loaded PLGA Nanoparticles as a Sustained Delivery System

**Published:** 2017

**Authors:** Saieede Soltani, Parvin Zakeri-Milani, Mohammad Barzegar-Jalali, Mitra Jelvehgari

**Affiliations:** a *Drug Applied Research Center, Tabriz University of Medical Sciences, Tabriz, Iran. *; b *Liver and Gastrointestinal Diseases Research Center, Tabriz University of Medical Sciences, Tabriz, Iran.*; c *Department ofPharmaceutics, Faculty of Pharmacy, Tabriz University of Medical Sciences, Tabriz, Iran.*

**Keywords:** Ketotifen fumarate, Nanoparticles, PLGA, Release

## Abstract

Ketotifen fumarate is a non-bronchodilator anti-asthmatic drug which inhibits the effects of certain endogenous substances known to be inflammatory mediators, and thereby exerts antiallergic activity. The present study describes the formulation of a sustained release nanoparticle (NP) drug delivery system containing ketoftifen, using poly (D,L lactide-co-glycolide) acid (PLGA). Biodegradable NPs were prepared using 50 : 50 PLGA by a water in-oil-in-water (w/o/w) double emulsion-solvent evaporation procedure and characterized for drug content, DSC (differential scanning calorimetry, XRD (X-ray diffractionl), FTIR (Fourier transform spectroscopy), particle size , surface morphology using scanning electron microscopy, and drug release rate. The effects of different drug-to-polymer ratios on the characteristics of the NPs were investigated. NPs prepared were spherical with a smooth surface. Size of NPs was dependent on the concentration of polymer (10 mg/mL, 754.6 nm). Increasing the external organic phase volume (primary emulsion) resulted in larger particles with higher encapsulation efficiency (55%). The best drug to polymer ratio in the NP was F_3_ (1:10 ratio) which showed loading efficiency of 55%, and mean particle size of 754.6 nm, respectively. The FTIR, XRPD, and DSC results ruled out any chemical interaction between the drug and PLGA. The NPs prepared with low ratio of drug to polymer (1:5) F_1_ formulation showed faster dissolution rate than those with high drug to polymer ratio (1:10) F_3_ formulation. In conclusion, by selecting an appropriate level of the investigated parameters, spherical NPs with encapsulation efficiencies higher than 55% and a prolonged drug release over 24h (73.67-90.05%) were obtained.

## Introduction

Ketotifen fumarate (KF) is a non-bronchodilator anti-asthmatic drug which hinders the effects of determined endogenous substances known as inflammatory mediators, and thereby poses antiallergic activity. KF possesses a powerful and sustained non-competitive histamine (H _1_) blocking property. To increase the bioavailability of the drug in the target site nanocarriers was developed as new drug delivery systems (1). 

Several nanocarriers such as nanoparticles (NPs), nanosuspensions, nanomicelles, liposomes, and dendrimers have been developed for various drug deliveries. As colloidal dispersions of nanosized drug particles, nanosuspensions are stabilized by surfactants. They can also be defined as biphasic systems consisting of pure drug particles dispersed in an aqueous vehicle in which the diameter of suspended particle is to be less than 1 μM (2). Nanosuspensions may be applied in enhancing the solubility of drugs that are poorly soluble in aqueous and lipid media; thereby, increasing the rate of flooding of the active compound and reaching the maximum plasma level rapidly. This is one of the unique advantages that nanosuspensions hold over other approaches for enhancing the solubility. It is useful for molecules with poor solubility, poor permeability or both, to pose a significant challenge for the formulators. Nanosuspensions differ from NPs (3), which are polymeric colloidal carriers of drugs (nanospheres and nanocapsules) to solid-lipid NPs (SLN), which are lipidic carriers of drugs (4).

A pharmaceutical nanosuspension is defined as a “very finely dispersed solid drug particle in an aqueous vehicle, stabilized by surfactants, for either oral and topical use or parentral and pulmonary administration, with reduced particle size, leading to an increased dissolution rate and therefore improved bioavailability". The diameter of the suspended particle is less than 1 μM (i.e. 0.1nm-1000 nm) (5-7). An increase in the dissolution rate of micronized particles (particle size < 10 μM) could be linked to an increase in the surface area and consequently the dissolution velocity. Nano-sized particles can increase the dissolution velocity and saturation solubility because of the vapor pressure effect (8, 9).

In addition, the diffusional distance on the surface of drug NPs is decreased; thereby leading to an increased concentration gradient. The increases in surface area and concentration gradient lead to a much more pronounced increase in the dissolution velocity as compared to a micronized product. These systems may render advantages in improving the rate and extent of absorption, and thus result in improved bioavailability (10). A nanoparticulate drug delivery system, once designed, can be evaluated on the basis of four important performance metrics. These are: particle size, drug incorporation efficiency, drug content, and drug release characteristics. Thus, a deeper understanding of all the factors monitoring the above mentioned performance metrics is of crucial importance in designing or planning a nanoparticulate drug delivery system for a particular drug.

PLGA or poly (lactic-*co*-glycolic acid) is a copolymer which is used in a host of Food and Drug Administration (FDA) approved therapeutic device, on account of its biodegradability and biocompatibility. PLGA has been successful as a biodegradable polymer because it endures hydrolysis in the body to produce the original monomers, namely, lactic acid and glycolic acid. These two monomers under normal physiological conditions are by-products of varied metabolic pathways in the body. Since the body effectively deals with these two monomers, there has been recorded minimal systemic toxicity as the result of PLGA utility in the drug delivery or its biomaterial applications (11, 12).

We have produced KF-loaded PLGA NPs using an emulsification solvent evaporation method and removed the non-encapsulated KF (free KF) from the formulation. Subsequent to production, the particles were extensively studied for factors influencing KF incorporation, particle size, and KF release from the particles. 

In the present investigation, KF was incorporated in the PLGA nanosuspension by using W_1_/O/W_2_ evaporation solvent emulsion method, with the aim of improving the loading efficiency.

*Materials and methods*

*Materials*

The PLGA polymer Resomer® 502 H (MW 7000-17000) was purchased from Sigma-Aldrich (Sigma-Aldrich Co. US). KF was supplied by Behansar Co. (Iran). Polyvinyl alcohol (MW 72000), dichloromethane (DCM), and sodium chloride were obtained from Merck (Germany). All solvents and reagents were of analytical grade.

*Experimental methods*

*Preparation of ketotifen-loaded PLGA NPs*

**Figure 1:**
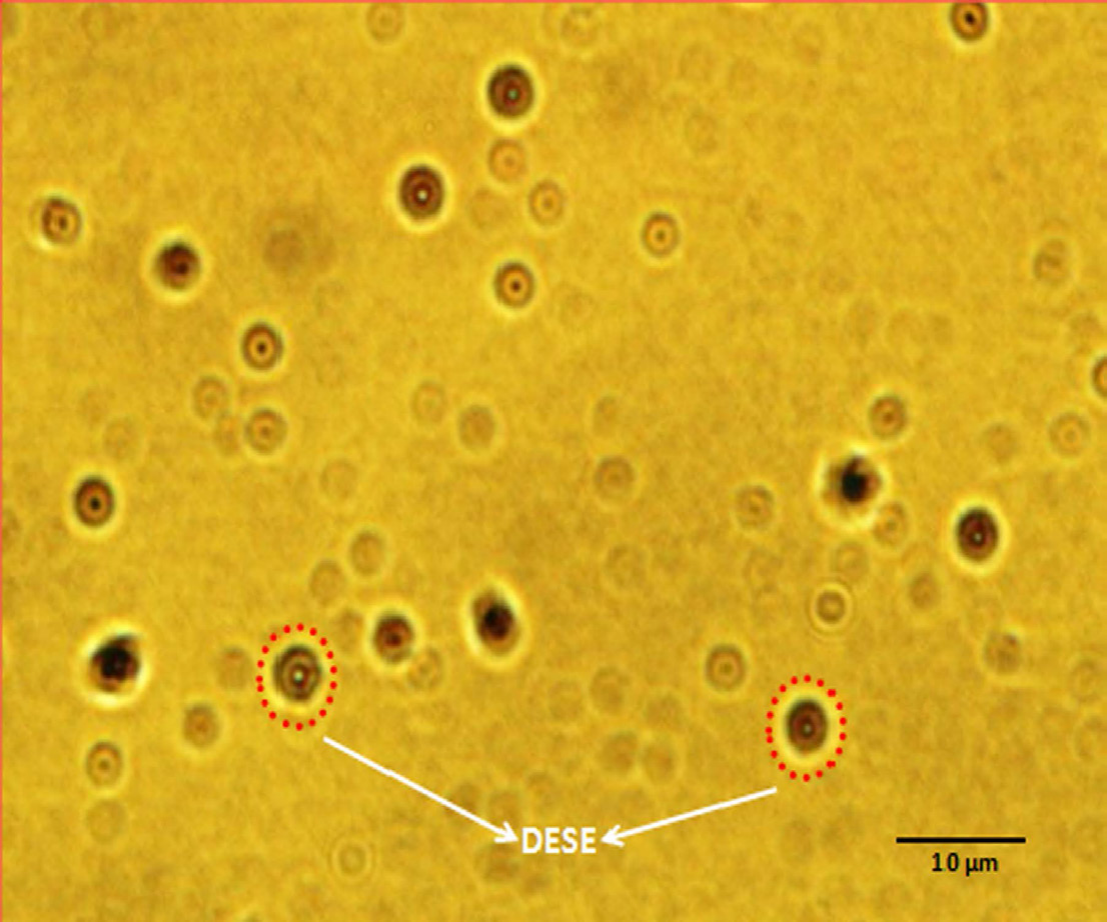
Light Microscope Image of double-emulsion solvent evaporation (W_1_/O/W_2_).

The NPs were prepared by double emulsion solvent evaporation technique (W_1_/O/W_2_) using different ratios of drug to polymer (1:5, 1:7.5 and 1:10) (Table1). An aqueous 0.5 % w/v KF solution (2 mL) was emulsified in 10 mL PLGA in DCM solution by using an ultrasound probe (Hielscher, UP200H, amplitude 80%) in an ice bath for 3 min. The W/O emulsion was added to 25 mL of water containing PVA (1% w/v) and NaCl (0.8% w/v) was sonicated for more 3 min to obtain W/O/W emulsion (Figure 1). Then this emulsion was diluted in 50 mL distilled water. The organic solvent was allowed to evaporate at room temperature under magnetic stirring (750-1000 rpm). Hardened NPs were collected and washed by centrifugation (Eppendorf, Centrifuge 5810 R, Germany) at 12000 rpm, 4 °C for 60 min. Supernatants were discarded and collected NPs were washed twice with ultra purified water (UPW) then resuspended in water and cooled down to -18 °C and freeze dried.

*Physicochemical characterization of (NPs)*

*Determination of drug loading and encapsulation efficiency of NPs*

In NPs, the external aqueous solution was obtained after centrifugation of the colloidal suspension for 60 min at 18,500 g. A standard calibration curve was performed with the KF solution (aqueous solution of 1% PVA).

Drug loading and encapsulation efficiency (EE%) were determined indirectly by UV spectrophotometry analyze of unincorporated drug in the supernatant of centrifuged nanosuspension at wavelength 298 nm. PVA 1% solution was used as blank solution. The loading of KF into PLGA NPs and encapsulation efficiency were calculated using following equation:

*Drug Loading (%) = (Total amount of drug-unincorporated drug amount)/ Amount of NP recovered ×100*

KF entrapment efficiency was expressed as the ratio of the KF amount measured in the supernatant to the total KF.

*EE (%) = (Actual drug content in NP/Total drug used in formulation) ×100*

*Percentage yield value*

The production yield of the NPs was determined by the accurate calculation of the initial weight of the raw materials and the last weight of the polymeric particles obtained. All experiments were performed in triplicate.

*NPs size and zeta potential analysis*

The size and zeta potential of freshly prepared nanosuspension were analyzed by Dynamic Light Scattering (Malvern, UK) using a Zetasizer. The analysis was performed at scattering angle of 90°, after appropriate dilution with distilled water. Each measurement was done in triplicate.

*Scanning electron microscopy*

The morphology of NPs was examined with a scanning electron microscope (SEM) (MIRA3 TESCAN, Czech Republic) operating at 15 KV. The samples were mounted on a metal stub with a double adhesive tape and coated with platinum/palladium alloy under vacuum.

*Evaluation of polymer-drug interaction*

Stability of PLGA entrapped KF NPs revealed by drug-polymer interaction was evaluated by using DSC, XRD, and FTIR studies. These techniques not only evaluate the drug -polymer interaction but also the physical state of polymer and drug in NPs. 

*Differential scanning calorimetry*

Differential scanning calorimeter (DSC) (Shimadzu, Japan) was used to monitor thermal events during heating. Samples weighing 5 mg were placed in open aluminium pans and heated from 25 to 300 °C at a rate of 10 °C/min and calibrated with alumina (Al2O3). DSC measurements were carried out on drug, PLGA, PVA and on different formulations. 

*X-ray powder diffractometry*

X-ray diffraction analysis was performed with the appa­ratus Bruker Axs, D8 Advance diffractometer with nickel-filtered CuKα radiation (a voltage of 40 KV and a current of 20 mA). The scanning rate was 2 ˚C/min over a 2θ range of 10 ˚C-90 ˚C and with an interval of 0.02 ˚C.

*Fourier transforms infrared spectroscopy*

A computerized fourier, which transforms infrared spectroscopy, FT-IR (Bruker, Tensor 27, USA) was used to obtain the spectra of various KF samples. The KBr discs were prepared with apply almost 2-3 ton tons of pressure for 2 to 5 min. The scanning range was 400-4000 cm^-1^ and the resolution was 1 cm^-1^. 

*In-vitro release studies*

The *in-vitro* release of drug from the polymeric NPs was studied by the dialysis bag diffusion technique and under sink conditions for all NPs formulations. 

The dialysis bag (cutoff 12,000 Da) retained NPs and allowed the diffusion of the drug immediately into the recipient compartment (water, pH 7.4) (13). A set amount of NPs (10 mg NPs/5 mL water) was immersed to 100 mL dissolution medium, preheated and maintained at 32 ± 1 °C in a water bath, then stirred at 50 rpm. 

Then 3 mL of the medium were withdrawn at preset times by an automatic sampling system (Erweka DT 70, Erweka GmbH, Heusenstamm, Germany), (0.25, 0.5, 1, 2, 3, 4, 6, 8 and 24 h). An equal volume of fresh medium was added after each sampling. The amount of KF in the release medium was determined by UV at 298 nm. Each experiment was performed in triplicate (14).

*In-vitro release kinetics*

The data obtained from release studies were fitted into kinetic models like zero order, first order, Higuchi, Hixson Crowell, Korsemeyer-Peppas, etc, to study the mechanism of drug release from the prepared NPs (15).

*Statistical analysis*

Where appropriate, release results were evaluated using a one-way ANOVA at 0.05 level of significance.

## Results and discussion

*Physicochemical properties of NPs*

A W/O/W multiple emulsion solvent evaporation method is mostly used for the encapsulation of water-soluble drug and therefore was the method chosen for the water-soluble KF drug. The emulsions are usually prepared by emulsifying the aqueous phase containing the drug, in polymeric organic solution. The Resulted w/o emulsion is added to second aqueous solution containing emulsifier. The organic solvent diffuses out of the polymer phase and into the aqueous phase, and is then evaporated forming drug-loaded polymeric NPs. At the end, the uniform-sized beads were collected (16). The organic solvent diffuses out of the polymer phase and into the aqueous phase, and is then evaporated forming drug-loaded polymeric NPs. At the end, the uniform-sized beads were collected (16).

In solvent evaporation method, the polymer was dissolved in an organic solvent such as DCM, which was also used as a solvent for the PLGA polymer. The mixed polymer and drug solution (w_1_/o) was then emulsified in an aqueous solution containing a surfactant or emulsifying agent to form an oil-in-water (o/w_2_) emulsion. After the formation of a stable emulsion, the organic solvent was evaporated either by reducing the pressure or by continuous stirring. The particle size was influenced by the type and concentration of stabilizer, ultrasound power, and the polymer concentration (17). In order to obtain small particle size, high-speed ultrasound or centrifugation could be employed (18).

In the case of hydrophilic drugs, a multiple w_1_/o/w_2_ emulsion needed to be formed with the drug dissolved in the internal aqueous phase. In the coacervation technique, the coating precipitates onto a droplet of the drug (18). Coacervation consists of three stages occurring under a constant agitation: (1) a solution must be formed with three immiscible phases: the core material (active ingredient), the coating material, and a solvent; (2) the liquid coating is deposited around the core material, which is achieved by mixing the coating phase with the solvent phase (in which the active ingredients reside); and (3) the coating is hardened thermally or by desolvation (19-21) 

Emulsion process produced PLGA spheres of 158-754.6 nm in diameter.

In the NPs provided through evaporation method, the amount of drug entrapped in the NPs was lower than the theoretical value. This represents that some free drug crystals were missed in the process of encapsulation out (21, 22). 

*Morphology of NPs*

**Figure 2:**
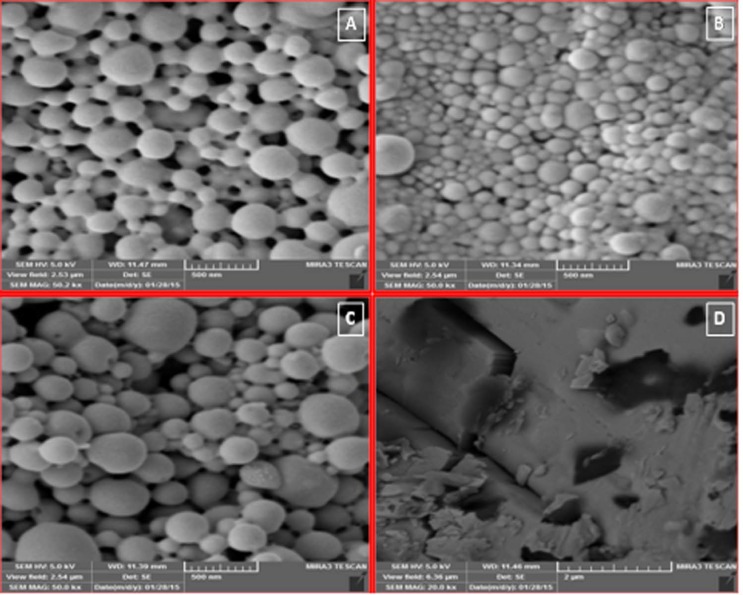
SEM images of ketotifen fumarate (KF) nanoparticles containing F_3_ (KF:PLGA) 1:10 ratio 50000x(A), F_3_ blank 50000x(B), F_2_ (KF:PLGA) 1:7.5 ratio 50000x(C), KF 20000x(D

Figure 2. shows SEM images of PLGA NPs. All preparations had homogenous distribution, spherical shape, and smooth surface characteristics. These spherical particles were in the range between 158 and 754.6 nm in size. They had high regular spatial arrangement and thus were described by an effective diameter. It has already been reported that particle size was proportional to the viscosity of the dispersed polymeric phase (O) . In fact viscosity of the dispersed phase was increased from F_1_ (1:5) to F_3_ (1:10). Particle size of NPs was directly proportional to the apparent viscosity of dispersed phase. When the dispersed phase with higher viscosity (F3 with 100 mg PLGA) was poured into the dispersion medium, bigger droplets were formed with larger mean particle size (754.6 nm).

*Influence of drug: polymer ratio on the physical properties of NPs*

Entrapment efficiency for different ratios of drug to PLGA polymer is shown in Table 2. The lowest EE% (43%) is related to the lowest polymer concentration and the highest one (55%) belongs to formulation with low ratio of drug to polymer (*p *< 0.05). The production yield of the NPs decreases (38.63%-67.68%) with the increase in the concentration of polymer (22). The yields of NPs as the function of PLGA concentration has been shown in Table 2. Although a satisfactory yield was obtained at PLGA lower concentrations (67.68%), the value decreased with the increase in the PLGA concentration. 

With DCM, the particles have a bimodal population distribution and, consequently, a high polydispersity of 0.83 (Table 2). Increasing the PLGA concentration in DCM from 5 mg/mL to 10 mg/mL changes the particle size distribution from unimodal to biomodal (Table 2). Light Microscope Image shows the effective particle diameters for a range of PLGA concentrations in DCM (Figure 1).

Bimodal distributions of particles were obtained with DCM at higher polymer concentrations. 

The polymer concentration and organic solvent selection plays a critical role in producing unimodal NPs. NPs are formed through a true emulsification mechanism conducted by DCM, the water immiscible solvent. In this mechanism, an external energy is applied to break the larger emulsion droplets into smaller ones (23). At higher polymer concentrations, the energy applied through ultrasound is insufficient to overcome the resistive viscous forces provided by the dissolved PLGA in the organic phase and the dissolved surfactant (PVA) in the aqueous phase leads to heterogeneous droplets and a bimodal size distribution.

The small polydispersity index suggested that the size distribution of the products is fairly monomodal. The above-mentioned monodispersed size distribution and excellent redispersability of NPs indicate that the surface of PLGA NPs is stabilized by some factors to prevent aggregation. Polydispersability was observed to be low, namely, in the range of 0.21 to 0.83. 

*Nanoparticles size and zeta potential analysis*

The particle size and zeta potential values are reported in Table 2. Particle size of formulations Fʹ_1_ (1:5) ratio and Fʹ_3_ (without sodium chloride) showed 889.6 and 935 nm, respectively. According of Table 2. for F_1_ and F_3_ formulations, presence of NaCl decreased the mean particle size significantly (F_1_ and F_3_ with 158 and 754.6 nm, respectively). Increasing the osmotic pressure of w_2 _(external phase of second emulsion) leads to water migration from w_1_ to w_2_ and a rapid shrinkage of the droplets. This phenomenon results in smaller nanoparticles (24). The most likely rationale to justify the findings might be the adsorption of PVA onto the surface of PLGA NPs. Process parameters such as PLGA and PVA concentration were determined to achieve the optimum processing conditions based on this manufacturing technique. This was probably caused by increasing the viscosity and hence resulting in poor dispersion of the PLGA solution in the aqueous phase. At higher concentrations of PLGA (10 mg/mL), decreasing the coacervation of PVA on the NP surface could be the leading cause of lower yields (38.63%). This phenomenon could occur as a consequence of worse dispersion of PLGA solution into aqueous phase owing to the increase of viscosity of PVA solution. This process led to the formation of uniform population of NPs having mean diameter in the range of 158 to 754.6 nm and also improved the loading efficiency as evident from 43% entrapment efficiency with 55%. The above mentioned monodispersed size distribution and excellent redispersability of NPs represents that the surface of PLGA NPs is stabilized by some factors to prevent aggregation. The most likely rationale to justify the findings might be the absorption of PVA on to the surface of PLGA NPs (25).

The zeta potential of an NP is commonly used to characterize the surface charge property of NPs. It shows that the electrical potential of particles is influenced by the composition of the particle and the medium in which it is dispersed. Currently principal technique involved in zeta potential determination is laser doppler anemometry. NPs containing zeta potential higher than (±) 30 mV have been demonstrated to be stable in suspension, since the surface charge hinders the aggregation of particles (26). These might lead to stronger repulsive interactions among the particles, and thus, higher stability of the particles is reached (27). Further, the zeta potential could be used to establish whether a charged active material is encapsulated within the center of the NPs or adsorbed onto the surface (26). To optimize formulation parameters and to make predictions as well, the zeta potential measurement is made, regarding the storage stability of the colloidal dispersion (28).

*Zeta potential*

Table 3 shows the values of the mean zeta potential of the prepared NPs. It was obvious that all formulae showed a surface negative charge with mean values ranging from -3.30 to -2.99 mV. The polymer type indicated that PLGA polymer had a negative value. This could be explained by the presence of free carboxylic acid end on the PLGA. It is worth mentioning that in case of PLGA the slightly negative charge on the surface may be attributed to the hydroxyl group of the PVA that was anchored on the surface of the NPs. The amphiphillic nature of the PVA leads to its entanglement in the polymer phase with their polar head group extruding out from the surface. This interaction is recorded to be strong and makes it difficult to be removed from the surface. Concerning the polymer mass and polymer ratio, it was shown that the higher polymer mass had a higher negative zeta potential as well as the higher polymer ratio (29). This might be explained by the significant increase in particle size due to the use of higher polymer mass and ratio thus increasing the exposed charge on the surface of particles.

**Figure 3:**
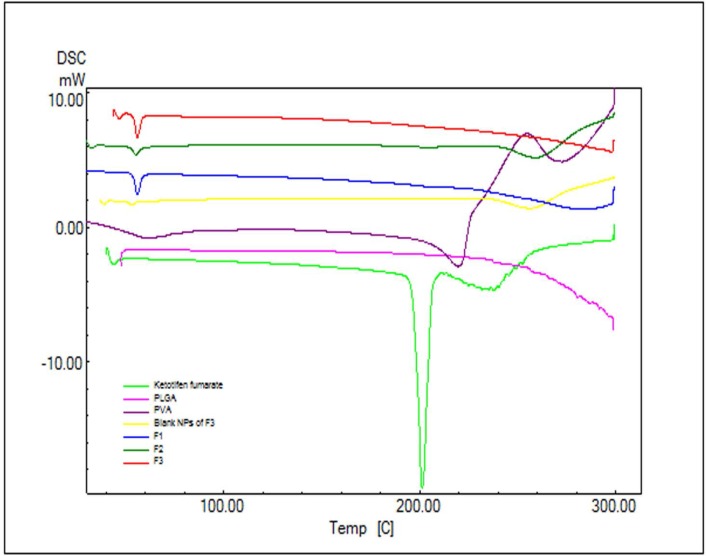
DSC thermogram of Ketotifen fumarate (KF); PLGA; PVA; blank NPs of F_3_; F_1 _(KF:PLGA) 1:5 ratio; F_2 _(KF:PLGA) 1:7.5 ratio; F_3 _(KF:PLGA) 1:10 ratio, respectively

**Figure 4:**
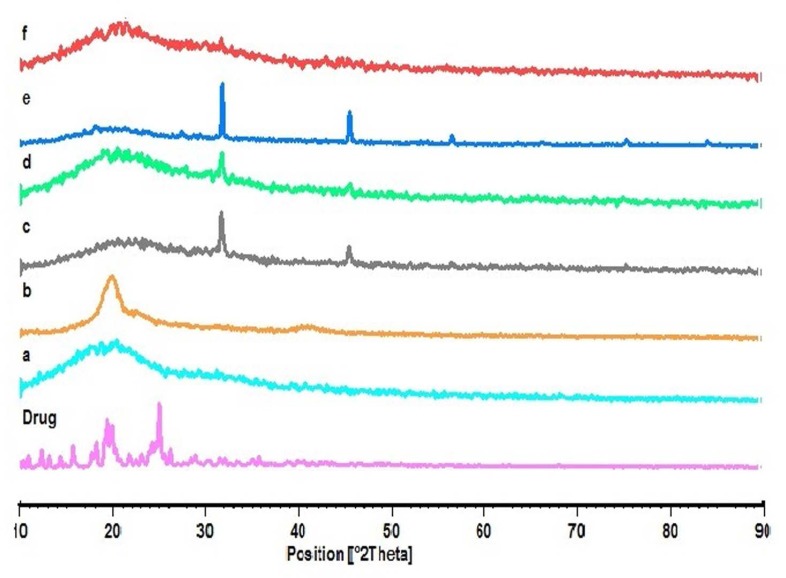
XRD thermogram of Ketotifen fumarate (Drug); PLGA (a); PVA (b); blank NPs of F_3 _(c); F_1 _(d); F_2 _(e); F_3 _(f), respectively

**Figure 5:**
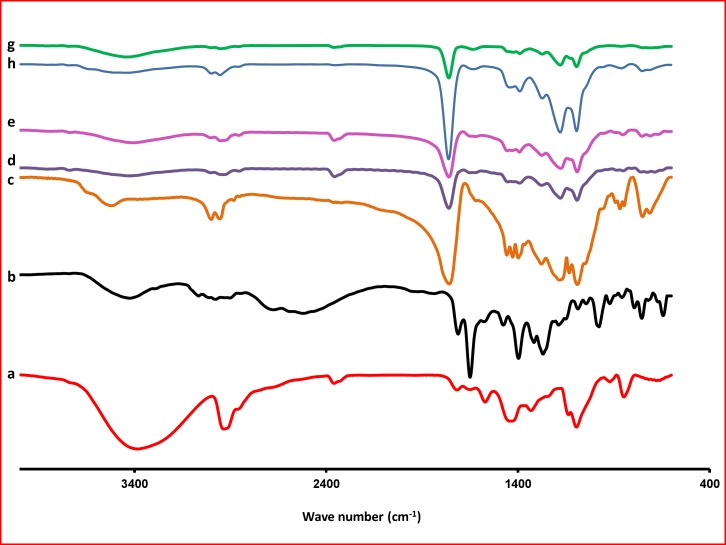
FTIR thermograms of PVA (a); KF (b); PLGA (c); F_1_ (KF:PLGA) 1:5 (d); F_2 _(KF:PLGA) 1:7.5(e); F_3_ (KF:PLGA) 1:10 (f); blank NPs of F_3 _(g); Physical mixture of F_3_(h), respectively

**Figure 6:**
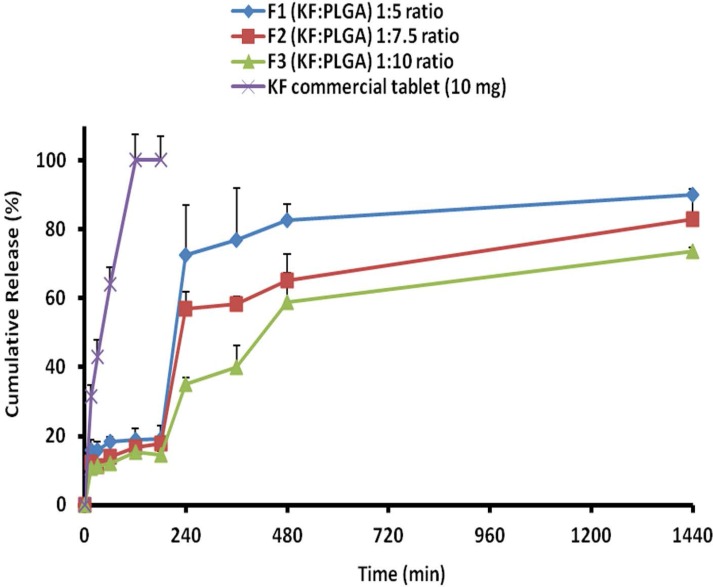
Cumulative percent release of KF from naoparticles with different polymers ratios and KF commercial tablet 10 mg

**Table 1:** ketotifen fumarate NPs prepared by double-emulsion solvent extraction (W_1_/O/W_2_

**Formulations**	**Drug:** **Polymer ratio**	**Initial emulsion (W**_1_ **/O)**				**Secondary aqueous phase** **(W**_2_ **)**
		**Initial aqueous** **phase (W**_1_ **)**		**organic phase** **(O)**		
		**Water** **(mL)**	a **KF** **(mg)**	b **PLGA** **(mg)**	c **DCM** **(mL)**	d **PVA (1%w/v)/** e **NaCl (0.8 %w/v)** **(mL)**
F_1_	1:5	2	10	10	10	25
F_2_	1:7.5	2	10	10	10	25
F_3_	1:10	2	10	10	10	25

a Ketotifen fumarate;

b Poly(lactic-*co*-glycolic acid);

c Dichloromethane;

d Polyvinyl alcohol;

e Sodium chloride.

**Table 2:** Effect of drug: polymer ratio on drug loading efficiency, production yield and particle size of ketotifen fumarate nanoparticles

**Formulation code**	**Drug : Polymer ratio**	**Production ** ** yield ** **(%±SD)**	**Theoretical** **drug content (%)**	**Mean** **drug entrapped** **(%±SD)**	**Drug loading** **efficiency** **(%±SD)**	**Mean particle** **size** **(nm)**
F_1_	1:5	67.68±2.11	16.67	10.58±0.85	43.00±8.00	158
F_2_	1:7.5	40.00±3.14	11.76	13.24±0.52	45.60±5.00	541.5
F_3_	1:10	38.63±2.19	9.10	4.07±0.23	55.00±12.00	754.6
*blank NPs of F_3_	-	40.00±2.47	-	-	-	237

* Blank NPs of F_3_ (without drug) were prepared under the same conditions without drug.

**Table 3:** Effect of drug to polymer ratio of ketotifen fumarate on the zeta potential and polydispersity index of the nanoparticles

**Samples**	**Zeta Potential** **(mV±SD)**	**Polydispersity Index** **(±SD)**
Ketotifen fumarate F_1_ F_2_ F_3_ Blank NPs of F_3_	6.68±0.00 -3.30±3.21 -3.19±8.21 -2.99±1.72 -19.2±0.00	0.68±0.00 0.21±0.29 0.51±0.00 0.83±0.06 0.25±0.00

**Table 4:** Comparison of various release characteristics of KF from different NPs formulations and commercial^® ^tablet

**Formulation**	a **Rel**_0.25_ **(%)**	b **Rel**_24_ **(%)**	c **DE**	d **T**_50%_ **(min)**	e **f**_1_
F_1_ F_2_ F_3_ KF commercial Tab^®^	15.81±3.04 12.30±0.52 10.67±0.26 31.49±3.24	90.05±1.60 86.40±7.42 73.67±1.18 100.36±2.22	74.49 62.72 54.04 97.20	248.84 352.61 383.74 45.47	44.68 54.72 63.38 0

a Rel_0.25_ = amount of drug release after 0.25 h;

b Rel_24_ = amount of drug release after 24 h;

c DE = dissolution efficiency;

d t 50% = dissolution time for 50% fractions;

e f_1_ = Differential factor (0<f_1_<15).

**Table 5:** Fitting parameters of the *in-vitro* release data to various release kinetics models

**Formulation**	**ORDER**	**MPE%**	**RSQ**	**k**	**n**	**Slope**	**Intercept**
F_1_	Peppas	2.58	0.882	0.122	0.090	0.090	-2.102
F_2_	Non conventional order 2	48.94	0.878	0.000	1.143	0.000	0.028
F_3_	Non conventional order 2	28.55	0.907	0.000	1.143	0.000	0.018
KF commercial Tab^®^	Non conventional order 2	0	1	0.0941	0.446	0.446	-2.363

*Differential scanning calorimetry analysis*

In order to study the crystalline or amorphous nature of formulations and to evaluate the interactions between drug, polymer, and other materials, DSC experiments were carried out (Figure 3). According to Figure 3. pure PLGA exhibits amorphous nature of PLGA polymer and no endotherms was observed in PLGA thermograms. The pure KF demonstrated a sharp peak at 201.24 °C which can be related to its melting point (Figure 3). The absence of a melting transition phase event can also be observed in the analysis of DSC curves. It can be inferred that all the analyzed copolymers are amorphous, which is in agreement with the literature. 

The DSC curve of NPs did not show the endothermic peak of KF. This suggests that the drug is incorporated into the NPs in a disordered and amorphous shape. Any severe alteration in the thermal behavior of either the polymer or the drug may be related to the drug-polymer interaction (30). In the thermogram of the PLGA-based NPs, there was a small endothermic peak at 55.27-56.14 °C which corresponds to the phase transition of PVA (Figure 3). Attending DSC thermograms, it is evident that the DSC curves of all NPs formulations are almost the same. In these curves, the peak of drug did not appear. This indicates that the KF might be dispersed/dissolved molecularly in the fused PLGA during the preparation of NPs (31). Based on thermodynamic calculations on the enthalpy of this endothermic, considering the heat flow of PVA in NPs and blank NPs of F_3_ (27.55, 13.44, 28.69 and 4.77 mJ for F_1_, F_2_, F_3_ and Blank F_3_ formulations respectively), the estimated loading of PVA in F_1_, F_2_, F_3_ and blank F_3_ formulations was calculated to be 26.29%, 12.83%, 27.38% and 4.55%, respectively.

*X-ray diffraction analysis*

The X-ray diffraction patterns reveal that the pure drug is crystalline in nature (Figure 4). However, when it was incorporated into the polymer matrix, the main peaks of the drug disappeared. This could be attributed to the amorphous state of the drug in the NPs. When the NPs are prepared with varying drug to polymer ratios (F_1_, F_2_ and F_3_), it is obvious that the NPs with lower polymer concentration would show similar peaks as the blank NPs. Some of the identifying peaks for KF are detectable at high concentration of polymer; though these peaks hold very low intensity due to the presence of lower concentration of drug in the sample compared to pure KF sample. This supports the results obtained from DSC.

*Fourier transform spectroscopy analysis*

The Fourier transform IR spectrum of KF alone showed that the principal peaks were observed at wave numbers stretching vibration N-H at 3424.64 cm−^1^, aromatic stretching vibration C=C at 1649.70 cm−^1^, bending vibration CH3 at 1476.99 cm-^1^, bending vibration phenolic OH at 1397.14 and CH out of plane bending vibrations in substituted ethylenic system (-C=CH- (cis) at 754.15 cm^-1^ (Figure 5). The spectra obtained by FTIR for the PLGA are presented in Figure 5. One can observe, in the spectra, the strong bands in the region between 1760 and 1750 cm^–1^, due to stretch of the carbonyl groups presented in the PLGA. There are also stretching bands due to asymmetric and symmetric C-C(=O)-O vibrations between 1300 and 1150 cm^–1^. The bands in these regions are of benefit in the characterization of esters. The 3525 and 3459 cm^–1^ bands in the FTIR spectra for lactide and glycolide are ascribed to moisture in the sample (OH group). These spectra corroborate those given in the literature for PLGA copolymers (32). The absorption bands between 3600 and 3400 cm^–1^ in the spectra presented in Figure 5, showing the hydroxyl group, indicates that the PLGA copolymers are hydrous.

In the FTIR, spectra of the blank NPs of F_3_ were observed at wave numbers 3436, 1651, 1477, 1396, and 756 cm^-1^. FTIR studies showed characteristic peaks of KF, confirming the purity of drug. FTIR spectral studies indicated there was interaction between KF and polymers used. 

For NPs, stretching vibration N-H are seen at 3400-3423, stretch of the carbonyl groups at 1760, asymmetric and symmetric C-C(=O)-O vibrations at 1390 and bending vibrations in substituted ethylenic system (-C=CH- (cis) at 725-752 cm^−1^. Differences in the positions of the absorption bands of KF were observed in spectra of the prepared formulations, indicating that there are chemical interactions in the solid state between the drug and the polymer.

*Release study*

The release behavior of KF from PLGA NPs, illustrated in Figure 6. indicates a biphasic pattern. Drug release profiles displayed a burst release in the first hour, followed by a lag phase and then sustained release appeared with the NPs. The drug release was more slowly occurred at a later stage, the rate of which might be controlled by the degradation speed of the polymer of NPs. It is worth noting that the degradation of polymer did not essentially occur, at least for the controlled period in the current system. KF encapsulated within PLGA, showed an initial burst of 10.67%-15.81% during the first 15 min, followed by a sustained release and until 24 h (73.67%-90.05), when most of the drug was (Table 4). The primary burst might be the result of rapid release of drugs deposited on the surface and in the water channels in NPs, as the mass of the polymer did not decrease during this period.

When NPs were kept in a release solution, the PLGA was dissolved slowly to form holes or disintegrate to release the drug. Principally, the concentration of the polymer induces this release of drug from the NPs . The compact nature of the polymer coat around the drug sustains the drug release. Particle size is another factor which strongly affects the rate of dissolution and solubility. It is believed that the particle size is inversely proportional to rate of dissolution and hence a higher rate of dissolution (F_1_>F_2_>F_3_ formulations) was observed with the developed NPs (33, 34).

The drug was very gradually released at a later stage, the rate of which was determined by the diffusion (n < 0.5) of the drug in the inflexible matrix structure. The values of dissolution models suggest that the drug release occurs mainly through diffusion processes. Peppas and non-conventional order 2 kinetics models (Table 5) shows the highest correlation; as it is evident from the values of regression coefficients (R^2 ^) for F_1_, F_2_ and F_3_ NPs as 0.882, 0.878, and 0.907, respectively (35).

*Conclusion*

The present work showed the KF-containing NP formation by the double-emulsion solvent evaporation method. It demonstrated the potential process for controlling the size of PLGA NPs. The NP formation process was to be associated with the reduction of globule size due to the rapid evaporation of solvent and hence NPs of particle size were produced. 

The size of droplets formation during the stage is an important factor. Variables such as drug to polymer ratio were found to be an important factor for the formation of PLGA NPs. Especially, the selected solvent such as DCM was used to dissolve the drug and significantly enhance the encapsulation of KF. PLGA encapsulated KF NPs yielded spherical shapes based on SEM results. The analysis of DSC and XRD patterns introduced well encapsulated compatible NPs as a drug carrier system. Release kinetics of KF, used as a model drug, showed sustained biphasic drug release governed by diffusion. The obtained results indicate the potential use of NPs accompanied with PLGA for the sustained release of KF. Optimization of formulations with different polymers and ocular applications are the future scope of study.

## References

[1] Fahmy RH, Badr-Eldin SM (2013). Novel delivery approach for ketotifen fumarate: dissofilms formulation using 32 experimental design: in-vitro/in-vivo evaluation. Pharmaceu.dev Tech..

[2] Arunkumar N, Deecaraman M, Rani C (2009). Nanosuspension technology and its applications in drug delivery. Asian J. Pharmaceu..

[3] Üner M (2006). Preparation, characterization and physico-chemical properties of solid lipid nanoparticles (SLN) and nanostructured lipid carriers (NLC): their benefits as colloidal drug carrier systems. Die Pharmazie-An Int. J. Pharmaceu. Sci..

[4] Üner M, Yener G (2007). Importance of solid lipid nanoparticles (SLN) in various administration routes and future perspectives. Int. J. Nanomed..

[5] Yadav GV, Singh SR (2012). Nanosuspension: a promising drug delivery system. Pharmacophore.

[6] Yadav M, Dhole S, Chavan P (2014). Nanosuspension: a novel techniqus in drug delivery system. World J. Pharm. Pharmaceu. Sci..

[7] Mukesh D (2012). Nanosuspension technology for solubilizing poorly soluble drugs. Int. J. Drug Dev. Res..

[8] Chingunpituk J (2011). Nanosuspension technology for drug delivery. Walailak J. Sci. Tech. (WJST).

[9] Patravale V, Kulkarni R (2004). Nanosuspensions: a promising drug delivery strategy. J. Pharm. Pharmaco..

[10] Almeida AJ, Souto E (2007). Solid lipid nanoparticles as a drug delivery system for peptides and proteins. Adv. Drug Deliver Review.

[11] Kim D, Frank C (2006). Fabrication and use of biocompatible materials for treating and repairing herniated spinal discs. Google Patents.

[12] Astete CE, Sabliov CM (2006). Synthesis and characterization of PLGA nanoparticles. J. Biomater. Sci. Polymer Edition..

[13] Aksungur P, Demirbilek M, Denkbaş EB, Vandervoort J, Ludwig A, Ünlü N (2011). Development and characterization of Cyclosporine A loaded nanoparticles for ocular drug delivery: Cellular toxicity, uptake, and kinetic studies. J. Control. Rel..

[14] Loveymi BD, Jelvehgari M, Zakeri-Milani P, Valizadeh H (2012). Statistical optimization of oral vancomycin-eudragit RS nanoparticles using response surface methodology. Iran. J. Pharm. Res.: IJPR.

[15] Gadad A, Chandra PS, Dandagi P, Mastiholimath V (2012). Moxifloxacin loaded polymeric nanoparticles for sustained ocular drug delivery. Int. J. Pharmaceu. Sci. Nanotech..

[16] Engel R, Riggi S, Fahrenbach M (1968). Insulin: intestinal absorption as water-in-oil-in-water emulsions. Nature.

[17] Naik J, Lokhande A, Mishra S, Kulkarni R (2012). Development of sustained release micro/nanoparticles using different solvent emulsification technique: A review. Int. J. Pharm. Bio. Sci..

[18] Barenholz Y, Gibbes D, Litman B, Goll J, Thompson T, Carlson F (1977). A simple method for the preparation of homogeneous phospholipid vesicles. Biochem..

[19] Manca ML (2007). Chitosan and PLGA microspheres as drug delivery system against pulmonary mycobacteria infections. Tesi di dottorato.

[20] Nasti A (2008). Chitosan-based nanoparticles and microparticles: Università degli Studi di Napoli Federico II.

[21] Stevanovic M, Uskokovic D (2009). Poly (lactide-co-glycolide)-based micro and nanoparticles for the controlled drug delivery of vitamins. Cur. Nanosci..

[22] Nagarwal RC, Kant S, Singh P, Maiti P, Pandit J (2009). Polymeric nanoparticulate system: a potential approach for ocular drug delivery. J. Control. Rel..

[23] Legrand P, Lesieur S, Bochot A, Gref R, Raatjes W, Barratt G (2007). Influence of polymer behaviour in organic solution on the production of polylactide nanoparticles by nanoprecipitation. Int. J. pharmaceu..

[24] Bodmeier R, McGinity JW (1987). The preparation and evaluation of drug-containing poly (dl-lactide) microspheres formed by the solvent evaporation method. Pharmaceu. Res..

[25] Mahboubian A, Hashemein SK, Moghadam S, Atyabi F, Dinarvand R (2010). Preparation and in-vitro evaluation of controlled release PLGA microparticles containing triptoreline. Iran. J. Pharm. Res.: IJPR.

[26] Mahajan N, Sakarkar DD, Manmode A (2011). Preparation and characterization of meselamine loaded PLGA nanoparticles. Int. J. Pharm. Pharmaceu. Sci..

[27] Mosqueira VCF, Legrand P, Pinto-Alphandary H, Puisieux F, Barratt G (2000). Poly (D, L-lactide) nanocapsules prepared by a solvent displacement process: Influence of the composition on physicochemical and structural properties. J. Pharm. Sci..

[28] Singh BP, Menchavez R, Takai C, Fuji M, Takahashi M (2005). Stability of dispersions of colloidal alumina particles in aqueous suspensions. J. Colloid Inter. Sci..

[29] Heurtault B, Saulnier P, Pech B, Proust J-E, Benoit J-P (2003). Physico-chemical stability of colloidal lipid particles. Biomat..

[30] Yasarla LR, Ramarao BV (2012). Dynamics of flocculation of lignocellulosic hydrolyzates by polymers. Indus. Engin. Chem. Res..

[31] Lim S-J, Kim C-K (2002). Formulation parameters determining the physicochemical characteristics of solid lipid nanoparticles loaded with all-trans retinoic acid. Int. J. Pharmaceu..

[32] Jelvehgari M, Barar J, Valizadeh H, Shadrou S, Nokhodchi A (2011). Formulation, characterization and in-vitro evaluation of theophylline-loaded Eudragit RS 100 microspheres prepared by an emulsion-solvent diffusion/evaporation technique. Pharm. Dev. Tech..

[33] Erbetta CDAC, Alves RJ, Magalh Resende J, de Souza Freitas RF, De Sousa RG (2012). Synthesis and Characterization of Poly (D,L-lactide-co-glycolide) Copolymer. J. Biomater. Nanobiotech..

[34] Venkatesh DN, Baskaran M, Karri VVSR, Mannemala SS, Radhakrishna K, Goti S (2015). Fabrication and in-vivo evaluation of Nelfinavir loaded PLGA nanoparticles for enhancing oral bioavailability and therapeutic effect. Saudi Pharm. J..

[35] Alexander A, Tripathi D, Giri TK, Khan J, Suryawanshi V, Patel RJ (2010). Technologies influencing rapidly disintegrating drug delivery systems. Int. J. Pharm. Prof..

[36] Korsmeyer RW, Gurny R, Doelker E, Buri P, Peppas NA (1983). Mechanisms of solute release from porous hydrophilic polymers. Int. J. Pharmaceu..

